# Three-dimensional images of a pulmonary dominant truncus arteriosus before and after a novel repair

**DOI:** 10.21542/gcsp.2021.9

**Published:** 2021-04-30

**Authors:** Mohamed Nagy, Hatem Hosny, Ahmed Afifi, Abdelrahman Elafifi, Magdi H. Yacoub

**Affiliations:** 1 Aswan Heart Centre, Aswan, Egypt; 2 National Heart Institute, Giza, Egypt; 3 Imperial College London, London, United Kingdom

## Abstract

This paper documents, for the first time, the *in vivo* size, geometry, and function of the different components of this important subtype of truncus arteriosus (pulmonary dominant). Previous descriptions were based on examining formalin-fixed (collapsed) specimens, or descriptions during operations. It is hoped that this information can be of value in designing operative treatment as well as interpreting future sequential imaging, with the aim of optimizing the results of comprehensive repair.

## Introduction

Truncus arteriosus (TA) is a complex congenital anomaly characterized by the presence of a single arterial outlet from the heart, which gives origin directly to the systemic, pulmonary, and coronary circulations. The condition has been repeatedly classified by Collett and Edwards,^1^ Van Praagh,^2^ and more recently by Robert Anderson and colleagues.^3^ The latest classification described two types, aortic or pulmonary dominance. The latter corresponded to TA with interrupted aortic arch in previous classifications.^1,2^

We present detailed pre- and post-repair (using a novel technique) 3D images of all the component parts of a pulmonary dominant truncus arteriosus.

## Patient and Methods

A 1-year-old female patient presented to Aswan Heart Centre with the clinical and echo diagnosis of truncus arteriosus.

Pre- and post-operative multislice computed tomography (MSCT) was performed using a Siemens Somatom Definition AS 128 (Siemens, Erlangen, Germany). 3D-segmentation and measurements were performed on Mimics Innovation Suite (Materialise, Leuven, Belgium).

The details of the novel repair technique will be the subject of future communication. In short, the technique consists of transection of the arterial trunk above and below the origin of the pulmonary arteries, wide mobilization of the latter, creation of an autologous neo-right ventricular outflow tract (RVOT), and importantly, tailoring each component, with the aim of restoring the pattern of flow in the heart.^4^

## Results

### Ventricular size, shape, and function

**Figure 1.**
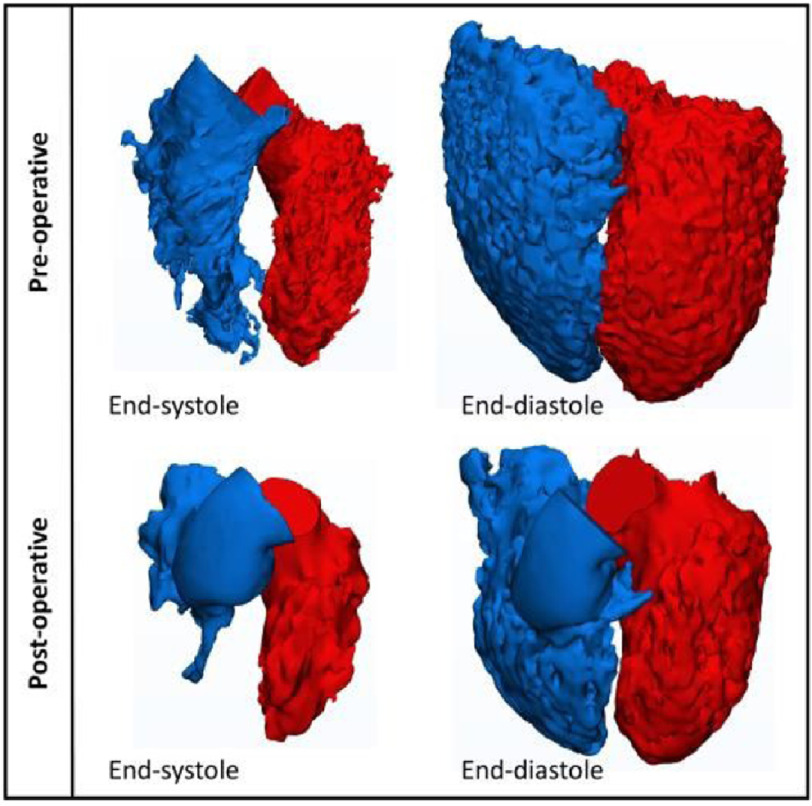
Pre- and post-operative 3D images of the cavities of both ventricles (in end-systolic and end-diastolic phases).

**Figure 2.**
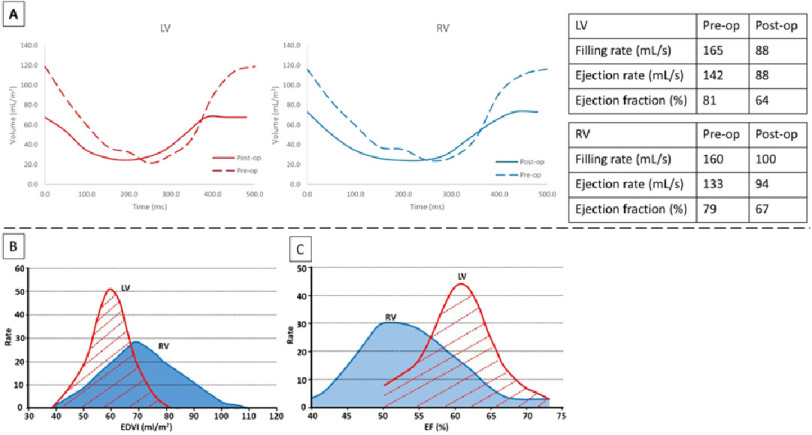
Pattern of emptying and filling of both ventricles (indexed volume per body surface area) before and after the repair. (A) Rate of distribution of indexed end-diastolic volume of both ventricles in healthy population (adapted from Sylva Kovalova et. Al.)^5^ (B) Rate of distribution of ejection fraction of both ventricles in healthy population (adapted from Sylva Kovalova et. Al.)^5^ (C).

Pre-operatively, the uncorrected truncus arteriosus results in a very large left-to-right shunt through the unrestrictive ventricular septal defect (VSD) as well as the communication between the trunk vessel and the pulmonary arteries. This results in much dilatation and hypertrophy of both ventricles (Figure 1). Post-operatively, separation of the systemic from the pulmonary circulation results in a considerable diminution of the volumes of both ventricles (Figure 1). This is accompanied by changes in instantaneous end-systolic and end-diastolic volumes during the cardiac cycle; as well as ejection fraction^5,6^ (Figure 2). These changes resulted in partial normalization of these parameters.^5^

### Origin of the arterial trunk from the ventricles

**Figure 3.**
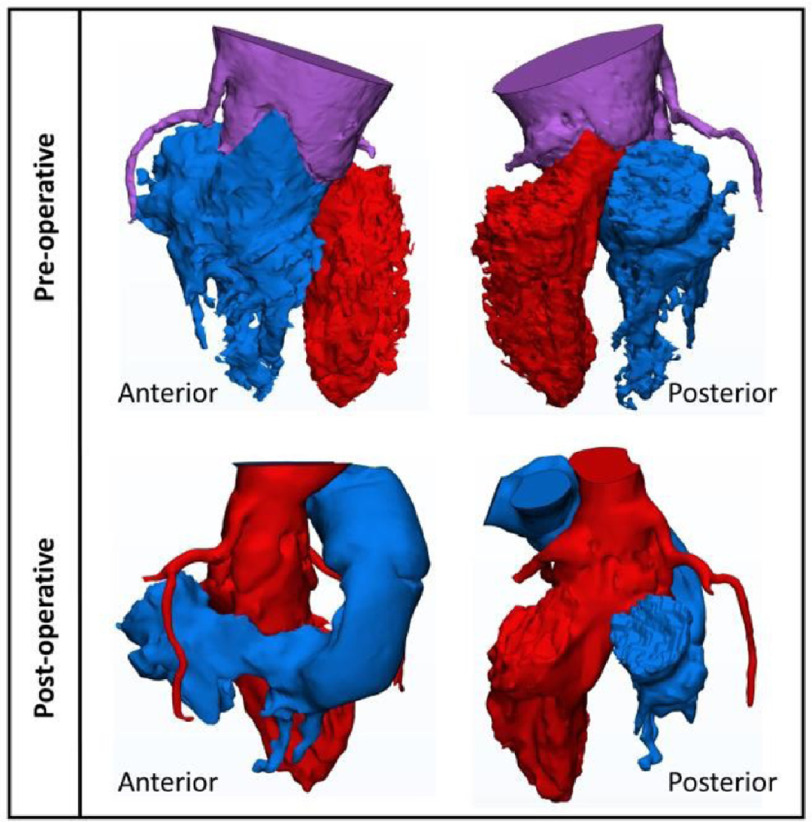
3D images of the ventriculo-arterial junctions before (upper row), and after repair (lower row), showing separation of the roots and origins of both coronary arteries.

**Figure 4.**
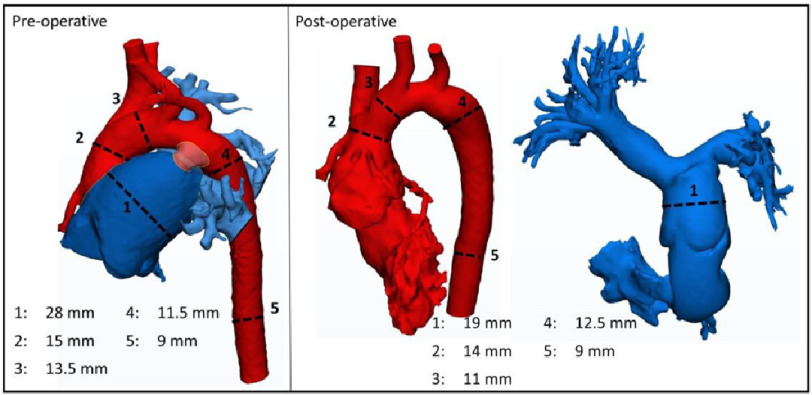
3D images of the pulmonary artery dominant truncus (dark blue) with the origin of the major systemic arterial system (in red) before repair, and after separation of the pulmonary from the systemic arterial system (neo-aorta). The patent ductus arteriosus (in pink) connecting the two systems before separation is also seen.

**Figure 5.**
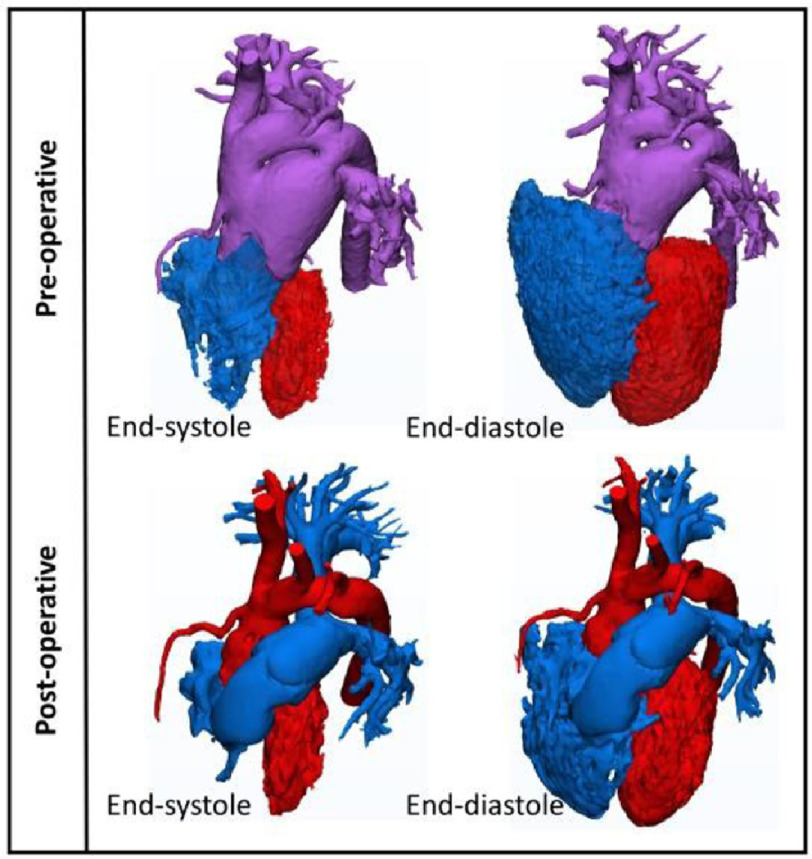
Overall appearance of all the components of the pulmonary dominant truncus before and after repair.

Before operating, the arterial trunk originated from both ventricles through a very large ventriculoarterial connection (Figure 3). Post-operatively, the neo-aorta arose from the left ventricle with a smaller normal-looking (Flask-shaped) neo-aortic root. The pulmonary artery is now connected to the right ventricle by a neo-RVOT as well as a 14 mm Contegra (Medtronic, Dublin, Ireland) valve with a preserved angle of pulmonary artery bifurcation (Figures 4 and 5).

### Coronary arterial origin and distribution

Pre-operatively, the right coronary artery arose from above the anterior sinus of the ventriculoarterial valve, while the left coronary artery arose from the posterior sinus (Figure 3). Postoperatively, the two coronaries arose from the anterior and posterior neo-aortic sinuses respectively (Figure 3).

### Mode of origin and communication of the great arteries

Before operating, the systemic vessel arose from the dominant pulmonary arterial trunk below the origin of the brachiocephalic artery with a slightly hypoplastic aortic arch (Figure 4). In addition, a five mm patent ductus in the region of the “isthmus” followed by slight dilatation of the descending aorta. After operating, there was a separation of the systemic from the pulmonary circulation with the tailoring of the neo-ascending aorta, division of the ductus, and refashioning of a neo-RVOT. The pulmonary artery is now connected to the right ventricle by a neo-RVOT as well as a 12 mm Contegra (Medtronic, Dublin, Ireland) valve with a preserved angle of pulmonary artery bifurcation.

### Overall appearance

This figure (Figure 5) shows all the changes in the individual components of the TA including the topology of the great articles, and the normal-like crossing appearance.

### Comments and future direction

This paper documents, for the first time, the in vivo size, geometry, and function of the different components of this important subtype of TA. Previous descriptions were based on examining formalin-fixed (collapsed) specimens or descriptions during operations. It is hoped that this information can be of value in designing operative treatment as well as interpreting future sequential imaging, with the aim of optimizing the results of comprehensive repair.

## Funding

This work was funded and supported by: Magdi Yacoub Global Heart Foundation and the Egyptian Science and Technology Development Fund (STDF).
